# Identification of an independent immune-genes prognostic index for renal cell carcinoma

**DOI:** 10.1186/s12885-021-08367-6

**Published:** 2021-06-29

**Authors:** Guangyao Li, Xiyi Wei, Shifeng Su, Shangqian Wang, Wei Wang, Yichun Wang, Xianghu Meng, Jiadong Xia, Ninghong Song, Chao Qin

**Affiliations:** 1 Department of Urology, the First Affiliated Hospital of Nanjing Medical University, Nanjing, 210029 China; 2 The Affiliated Kezhou People’s Hospital of Nanjing Medical University, Kezhou, Xinjiang, 845350 China

**Keywords:** Immune-related genes, Clear cell renal cell carcinoma, Prognosis, Nomogram, Immune risk score model

## Abstract

**Background:**

Considerable evidence has indicated an association between the immune microenvironment and clinical outcome in ccRCC. The purpose of this study is to extensively figure out the influence of immune-related genes of tumors on the prognosis of patients with ccRCC.

**Methods:**

Files containing 2498 *immune*-related *genes* were obtained from the Immunology D*atabase* and Analysis Portal (*ImmPort*), and the transcriptome data and clinical information relevant to patients with ccRCC were identified and downloaded from the TCGA data-base. Univariate and multivariate Cox regression analyses were used to screen out prognostic immune genes. The immune risk score model was established in light of the regression coefficient between survival and hub immune-related genes. We eventually set up a nomogram for the prediction of the overall survival for ccRCC. Kaplan-Meier (K-M) and ROC curve was used in evaluating the value of the predictive risk model. A *P* value of < 0.05 indicated statistically significant differences throughout data analysis.

**Results:**

Via differential analysis, we found that 556 immune-related genes were expressed differentially between tumor and normal tissues (*p* < 0. 05). The analysis of univariate Cox regression exhibited that there was a statistical correlation between 43 immune genes and survival risk in patients with ccRCC (*p* < 0.05). Through Lasso-Cox regression analysis, we established an immune genetic risk scoring model based on 18 immune-related genes. The high-risk group showed a bad prognosis in K-M analysis. (*p* < 0.001). ROC curve showed that it was reliable of the immune risk score model to predict survival risk (5 year over survival, AUC = 0.802). The model indicated satisfactory AUC and survival correlation in the validation data set (5 year OS, Area Under Curve = 0.705, *p* < 0.05). From Multivariate regression analysis, the immune-risk score model plays an isolated role in the prediction of the prognosis of ccRCC. Under multivariate-Cox regression analysis, we set up a nomogram for comprehensive prediction of ccRCC patients’ survival rate. At last, it was identified that 18 immune-related genes and risk scores were not only tremendously related to clinical prognosis but also contained in a variety of carcinogenic pathways.

**Conclusion:**

In general, tumor immune-related genes play essential roles in *ccRCC* development and progression. Our research established an unequal 18-immune gene risk index to predict the prognosis of ccRCC visually. This index was found to be an independent predictive factor for ccRCC.

**Supplementary Information:**

The online version contains supplementary material available at 10.1186/s12885-021-08367-6.

## Background

Renal cell carcinoma (RCC) is among one of the most prevalent malignancies affecting humanity worldwide. Its incidence rate has increased during the past 10 years, consisting of 2–3% of the whole newly diagnosed carcinoma cases [1]. In histology, clear cell RCC (ccRCC) is the predominant RCC subtype, responsible for nearly 75% of total renal carcinoma cases [2]. As considerable progress has been achieved in screening, diagnosing, and treating a variety of types of tumors through surgery and drug therapy [3–5], the clinical prognosis of ccRCC remains unsatisfactory [2, 6]. Thus, identifying several prognostic factors and targets is crucial to making the therapy and clinical outcomes of ccRCC patients better.

Immune evasion has recently attracted great interest as one of the fundamental characteristics of carcinoma [7]. Immunotherapies, including immune checkpoint blockade, have produced astonishing results in the management of malignancies. Accumulating evidence shows immune-related components, including immune genes, antigens, and immune cells, contribute greatly to the occurrence and malignant progression of cancer and are valuable markers for cancer diagnosis and prognosis [8]. Additionally, immune genes in tumor TME have great potential as prognostic biomarkers [9]. However, IRG predictive models still need extensive study when it comes to ccRCC biology.

This study aims to reveal the distribution and pedigree of immune-related genes in patients with ccRCC and explore the influence of immune-related genes on the prognosis of ccRCC cases. In addition, we established an immune genetic risk score model and set the nomogram, which was used for predicting the prognosis of ccRCC.

## Methods

### Data acquisition

First, through the ImmPort data-base, we gained the table of 2498 immune genes, then downloaded the transcriptome records. A total of 72 paracancerous tissues and 507 cancerous tissues were included in ccRCC cases from the TCGA data-base. Furthermore, the clinicopathological data of 507 ccRCC patients were gained as well, which include age, sex, pathological grading, tumor staging and TNM staging, vital status, and survival time (Table 1). At last, the correction of transcriptome records depends on the “LIMMA” software package in R software.

**Table 1:** Clinical characteristics of included patients in the study

Variables	Total (*n* = 507)	Training cohort (*n* = 252)	Validation cohort (*n* = 255)
Age (year)			
< 40	17	9	8
40–59	225	110	115
60–79	253	130	123
80+	22	13	9
Gender			
FEMALE	179	89	90
MALE	338	173	165
Grade			
G1	13	6	7
G2	223	125	98
G3	203	91	112
G4	73	37	36
GX	5	3	2
Stage			
I	257	136	121
II	55	25	30
III	123	63	60
IV	82	38	44
T stage			
T1	263	137	126
T2	67	31	36
T3	176	87	89
T4	11	7	4
N stage			
N0	236	124	112
N1	15	6	9
NX	266	132	134
M stage			
M0	414	217	197
M1	77	35	42
MX	26	10	16

### Function analysis of related genes

For exploring the principal biological processes of the selected hub genes, we conducted the Kyoto Encyclopedia of Genes and Genomes (KEGG) and gene ontology (GO) analysis. The enriched KEGG and GO terms were identified by DAVID (https://david.ncifcrf.gov/).

### Survival analysis of hub genes and comparison of their expression levels

RCC cases’ clinical records from the TCGA data-base include survival time, vital status, and TNM staging (remove missing information cases). The survival analysis of the hub genes was carried out by the survival R software package. A log-rank test was used to detect the difference in overall survival. Survival curves were demonstrated by using the Kaplan-Meier method. *P* < 0.05 was considered statistically significant.

### Gene functional-enrichment analysis

For studying the biological characteristics of renal carcinoma, we performed gene enrichment analysis (GSEA, version 3.0, the broad institute of MIT and Harvard, http://software.broadinstitute.org/gsea/downloads.jsp) between the cancerous tissues and paracancerous tissues. The number of permutations is 1000; collapse dataset to gene symbols is “false”, and permutation type is “phenotype”. Additional options selected included weighted enrichment statistics, and Signal2Noise metric was applied to ranking genes. The experimental group was composed of a high-expression set, and the control group was composed of a low-expression set. Gene set databases c2.cp.kegg.v7.0.symbols.gmt was applied to enrichment analysis. Cut-off criteria included gene set size > 500 and < 15, while nominal *P* value of < 0.05 and an FDR of < 0.25 were considered significant.

### Statistical analysis

The analysis was carried out entirely through R statistical language version 3.6.1 (https://www.R-project.org). All of the tests had two sides, and a level of *P* < 0.05 was accepted as statistically significant. The continuous variables following normal distribution were compared by independent t-test, while those in skewness were compared by Mann-Whitney U test. In the light of the Pearson correlation coefficient, correlation matrices were schemed using R-software. We study the connection between OS and immune cell infiltration on the basis of the Kaplan-Meier curve, assessed with a log-rank test. The relevance between OS and immune cell infiltration was visualized by the K-M curve and further evaluated by log-rank test. Sensitivity and specificity in the predictive model of recurrence were analyzed by time-dependent ROC curves. The univariate regression model was applied in analyzing the influence of single-variable on survival. The LASSO-Cox regression models were used to identify the independent factors for survival. According to the Cox analysis, we used regression coefficients to build a nomogram.

## Results

### Differentially expressed genes of ccRCC

**Fig. 1:**
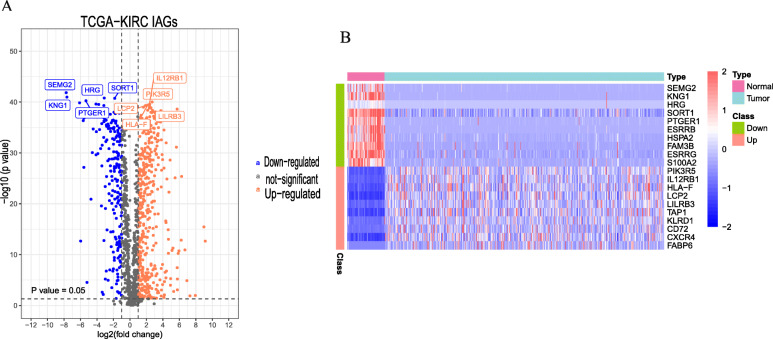
Differential gene expression profiles. **A** The volcano plot showed 540 differential expressions of genes in ccRCC and normal tissues based on TCGA data-base. **B** Heat map of the differentially expressed genes (topmost 10 upregulated and downregulated genes). The colors from green to red in the heat map represent a low-to-high level of expression. Red and green dots mean up-and down-regulated genes, respectively, and the black ones represent genes that are not differentially expressed. All the data and pictures were analyzed and then generated by R statistical language version 3.6.1 (https://www.R-project.org)

Files containing 2498 *immune*-related *genes* were obtained from the ImmPort data-base. For analyzing differential expression, transcriptome records of 72 para cancer and 507 tumor tissues were obtained from the TCGA data-base. By differential expression analysis between cancer tissues and normal tissues, 556 differentially expressed immune genes were recognized, among which 402 immune genes were upregulated and the remaining 154 immune genes were downregulated (*P* < 0.05, Fig. 1A). Heat map of the topmost ten up-and down-regulated differentially expressed genes are displayed in Fig. 1B.

### Functional annotation of differentially expressed genes in renal carcinoma

**Fig. 2:**
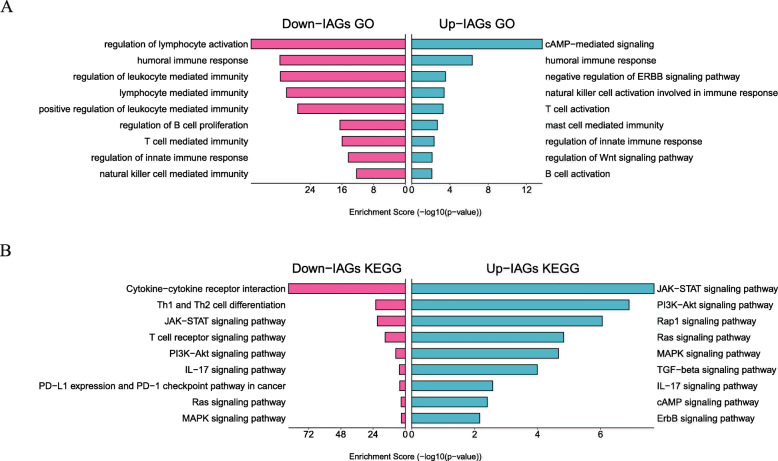
Gene Ontology (**A**) and Kyoto Encyclopedia of Genes and Genomes

We learned about the biological properties of 556 DEIGS by KEGG and GO analysis. David’s results showed that the topmost 3 enrichment GO items of the upregulated genes were cAMP-mediated signals, humoral immune response and negative regulation of ERBB signaling pathway, while the topmost 3 enrichment GO components of down-regulated genes are lymphocyte activation, humoral immune response, and regulation of leukocyte mediated immunity (Fig. 2A). Through pathway enrichment analysis, it was found that the top 3 biological pathways enriched with upregulated genes were the JAK − STAT signaling pathway, PI3K − Akt signaling pathway and Rap1 signaling pathway. In comparison, the top 3 biological pathways enriched with down-regulated genes were the cytokine-cytokine receptor interaction, Th1 and Th2 cell differentiation, and JAK-STAT signaling pathway (Fig. 2B).

### Construction of the immune-related prognostic model

**Fig. 3:**
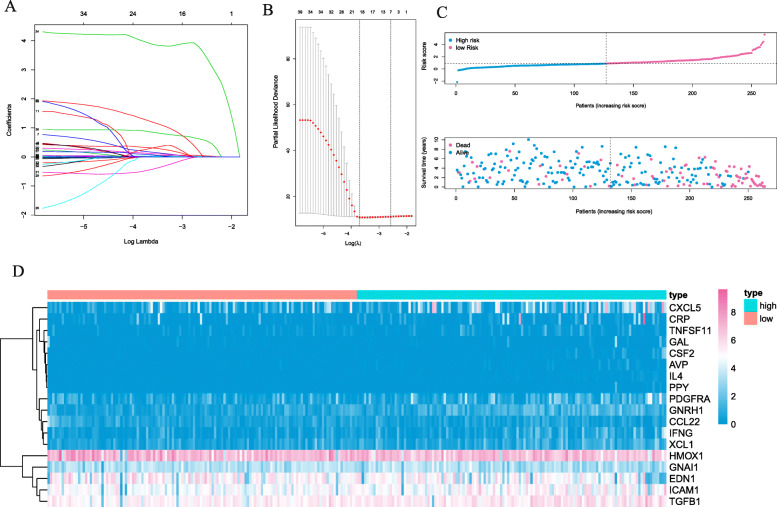
**A**–**B** The construction of the risk score model using the LASSO Cox regression model and 18 prognostic immune-related genes. **C** Distribution of immune-related risk scores and survival status in the training group. **D** Heatmap of model immune genes between the high-risk and low-risk sets (separated by median value) in the training group

**Fig. 4:**
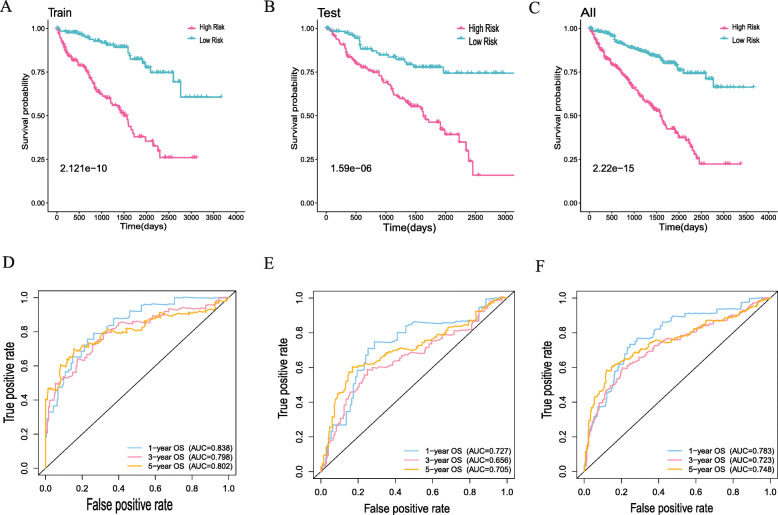
**A** K-M curve for analyzing high-and low-risk cases in the training group. **B** K-M curve for analyzing high- and low-risk cases in the test group. **C** K-M curve for analyzing of high- and low-risk cases in the whole TCGA group. **D** ROC curve, depending on time, for analyzing the training group. **E** ROC curve, depending on time, for analyzing the testing group. **F** ROC curve, depending on time, for analyzing the whole TCGA group

**Fig. 5:**
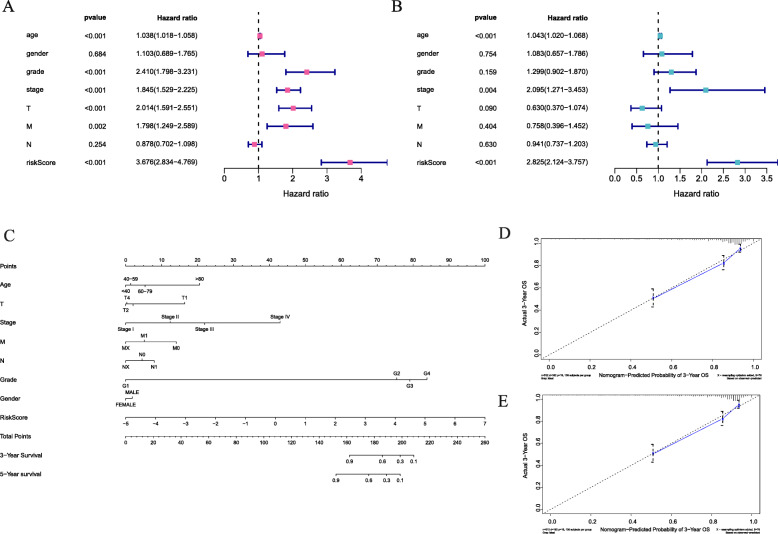
Cox proportional risk model for overall survival of related elements in ccRCC patients. **A**-**B** Univariate- and multivariate-Cox regressions analyses for 7 clinical prognostic factors influencing OS, respectively. **C** Nomogram for forecasting 3-year and 5-year prognosis of ccRCC. **D**–**E** Plots present the calibration curves used to compare the predicted and actual 3-and 5-year OS

We established a PPI network that was based on the differentially expressed genes and recognized 496 genes with over 50 para cancer nodes (Figure S1). To reveal relationships of these 496 DEIGs with the prognoses of patients with ccRCC, we identified 43 prognostic DEIGs through single variable Cox regression analysis (Table 2). KIRC data, downloaded from TCGA, were separated into 2 groups at random (training group: validation group, 1:1). After that, a lasso regression study was performed for the purpose of increasing robustness and selecting isolated indexes for survival in all according to the training group. At last, 18 DEIGs were obtained for the establishment of a prognostic indicator (Fig. 3A, B, Table 3). In the light of the risk index established, we divide patients into high-risk groups or low-risk ones (Fig. 3C). The differential expression of the model genes between high-and low-risk sufferers in the training set of ccRCC is shown in this heatmap (Fig. 3D). The Kaplan-Meier analysis indicated shorter overall survival among those high-risk sufferers in the validation and training set (*p* < 0.05, Fig. 4A, B, C). The ROC curve indicated better sensitivity and specificity in the risk model when used to predict survival risk (the AUC values of 5-year overall survival in the validation group and training validation group are 0.705 and 0.802, separately, Fig. 4D, E, F). In light of multivariate and univariate Cox regression analysis of age, sex, tumor staging, pathological grade, TNM stage and risk score, we determined if the immune risk score model was isolated of age, sex, tumor stage and other clinicopathological parameters. Among the single variable Cox models, age, pathological grading, tumor staging, T, M stage and high-risk scores are related to low survival rates (Fig. 5A). Of the multivariate Cox model, age, pathological grade and risk score were the only isolated predictors (Fig. 5B). For forecasting the prognosis of patients with ccRCC at 3 and 5 years after operations, we established another nomogram (Fig. 5C, D, E) based on the variables related to the overall survival rate ((OS)), namely age, sex, tumor staging pathological grade, TNM staging and risk score (Fig. 5C, D, E).

**Table 2:** Multivariate cox regression analysis to establish immune genes risk score model

Gene	Coef
ICAM1	0.0058648074324021
IFNG	0.0336285898183753
CXCL5	0.00449394306613633
XCL1	0.178774321649646
TGFB1	0.0114011479579204
PDGFRA	0.0346969626392538
GNAI1	0.00617576278056856
TNFSF11	0.321235808441014
HMOX1	−0.00150622701372233
CCL22	−0.456892844189334
IL4	3.92829889144972
CRP	0.00127583274886622
EDN1	−0.00254186826990028
AVP	1.2503911973052
CSF2	0.873478979686199
GAL	0.0818075259757682
GNRH1	0.1244306056583
PPY	0.275246400052544

**Table 3:** LASSO cox regression analysis to establish immune genes risk score model

Gene	Coef
ICAM1	0.0058648074324021
IFNG	0.0336285898183753
CXCL5	0.00449394306613633
XCL1	0.178774321649646
TGFB1	0.0114011479579204
PDGFRA	0.0346969626392538
GNAI1	0.00617576278056856
TNFSF11	0.321235808441014
HMOX1	−0.00150622701372233
CCL22	−0.456892844189334
IL4	3.92829889144972
CRP	0.00127583274886622
EDN1	−0.00254186826990028
AVP	1.2503911973052
CSF2	0.873478979686199
GAL	0.0818075259757682
GNRH1	0.1244306056583
PPY	0.275246400052544

### Correlation between 18 immune genes together with a risk score of immune gene and clinical prognosis

**Fig. 6:**
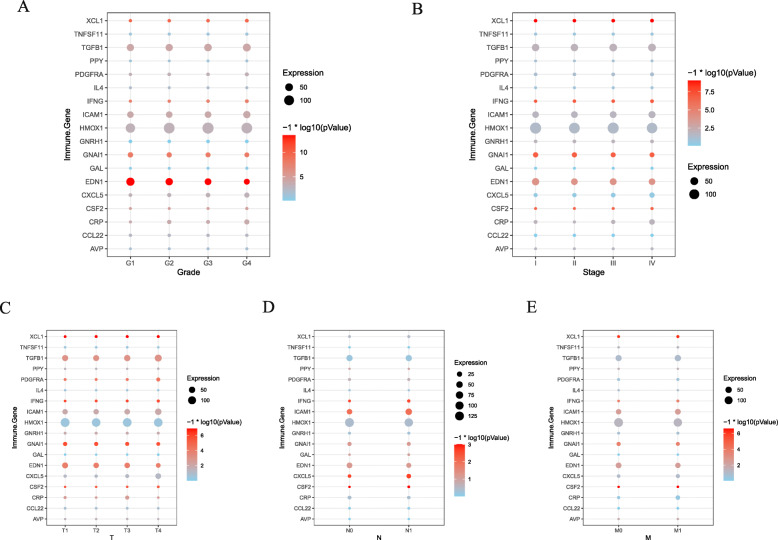
Correlation analysis of 18 immune genes with pathological grade, tumor stage and TNM in ccRCC patients. **A** Correlation between 18 immune genes and pathological grade of ccRCC patients. **B** Correlation between 18 immune genes and tumor staging of ccRCC patients. **C**-**E** Correlation of 18 immune genes with tumor, node, and metastasis classification in ccRCC patients

**Fig. 7:**
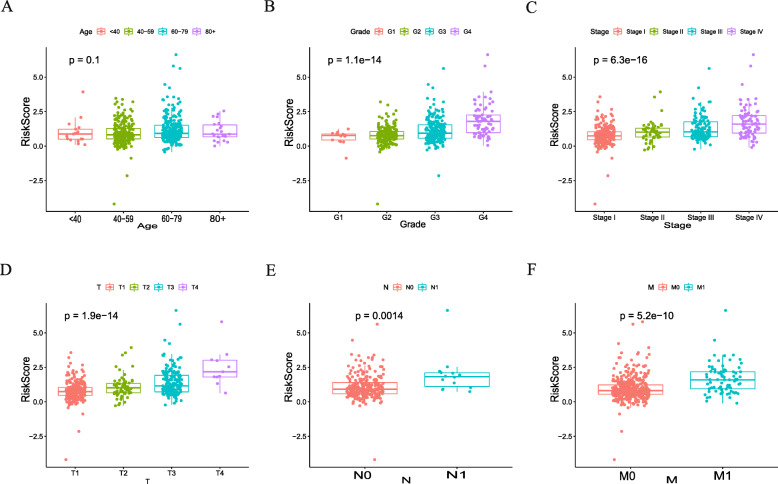
Correlation analysis of immune-related gene risk score and clinicopathological elements. **A** Age. **B** Pathological Grade. **C** Tumor Stage. **D** T. **E** N. **F** M

The scale of every gene model was determined at different pathological stagings. EDN1, GNAL1, and ICAM1 were remarkably correlated with the progression of ccRCC (Fig. 6). IFNG’s and XCL’s expressions were connected to the infiltration of CD4+, CD8+, and myeloid dendritic cells (Figure S2). The immune gene risk score was closely related to the grade, pathological stage, and clinical TNM stage (Fig. 7).

### Gene set enrichment analysis results with hallmark genes of risk scores

**Fig. 8:**
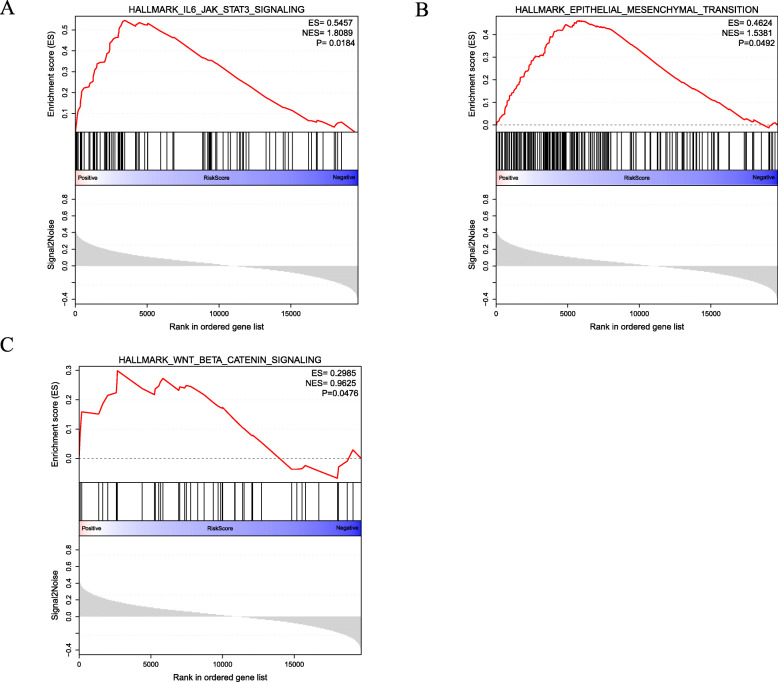
GSEA of the risk scores of immune genes

To investigate the biological connection of risk scores in ccRCC development, we carried out a GSEA on the risk scores from a TCGA renal carcinoma group. It showed that high-risk scores are connected to IL6 JAK STAT3 SIGNALING, EPITHELIAL MESENCHYMAL TRANSITION, and WNT BETA CATENIN SIGNALING (Fig. 8).

## Discussion

ccRCC is a heterogeneous disease with different ethnic characteristics resulting from renal epithelial cells [10] and accounts for most RCC-related deaths [11]. Although radical nephrectomy has been proven to be an effective treatment for local renal cancer. Many patients may experience development and metastasis after surgical resection. Given that targeted treatment for advanced and metastatic ccRCC has been fully developed, response to treatment is diverse [12]. Identification of molecular mechanisms and relevant prognostic factors may be critical to the treatment of ccRCC [13]. The prognosis of the tumor is closely connected with TME, particularly when considering the tumor immune microenvironment [14, 15]. Different types of cancers have diverse immune gene subpopulations. Therefore, investigating the immune gene subsets is vital for evaluating the risk and prognosis of ccRCC.

In the research, a large amount of specimen data was conducted to assess the immune genes of ccRCC in comprehensiveness and detail. We analyzed the expression of 2498 immune genes that were gained from the ImmPort database in ccRCC and normal tissues. Furthermore, we recognized and set up a risk score model for ccRCC via a single variable and LASSO-Cox regression analyses. The model was composed of 18 DEIGs (AVP, CCL22, CRP, CSF2, CXCL5, EDN1, GAL, GNAI1, GNRH1, HMOX1, ICAM1, IFNG, IL4, PDGFRA, PPY, TGFB1, TNFSF11 and XCL1). The K-M analysis showed that high-risk scores not only were associated with lower overall survival but also predicted advanced stage and higher pathological grade.

Several genes in the model have been studied in renal cell carcinoma. Arginine vasopressin (AVP) and its type 2 receptor (V2R) play an important role in regulating salt and water homeostasis. Activation of V2R can stimulate the proliferation of renal cell carcinoma (RCC) cell line in vitro [16]. It has been shown that the increase of CXCL5 cytokines is associated with sunitinib resistance in renal cell carcinoma [17]. CCL22, CRP, ICAM1, IFNG, PDGFRA, TGFB1 and TNFSF11 have been confirmed to be involved in the malignant progression and metastasis of renal cell carcinoma through different biological mechanisms [18–24]. EDN1 may serve as a promising prognostic and diagnostic biomarker for ccRCC [25]. In addition, CXCL5, IL4 may be involved in the regulation of the immune microenvironment in renal cell carcinoma [26].

ccRCC immune models are typically established by screening immune-related lncRNAs. A new prognostic gene marker based on immune lncRNA in patients with KIRC patients was found [27]. Zhao et al. integrated multiple levels of data to construct immune, inflammatory, or KIRC-oriented neighbor networks and KIRC-related gene directed networks. Their analysis showed that genes related to immune and inflammation have unique topological characteristics and related KIRC expression patterns in the networks. Furthermore, they identified five core clusters for constructing specific prognostic biomarkers for KIRC [28]. Another study evaluated the prognostic value of individual gene expression by using TCGA data and ccRCC patient data [29]. In the study, a predictive nomogram was generated. Independent prognostic factors were identified not only for examining the functional involvement of individual genes in vitro and in vivo RCC models but also for the assessment of OS and progression-free survival of patients with ccRCC in the first, fifth, and eighth year [29]. Our study shed light on the role of immune-related genes in tumorigenesis and malignant development of ccRCC. Based on immune genes, we established a novel risk-score model consisting of immune genes and verified it to predict ccRCC prognosis. Our risk model showed excellent predictive performance in terms of prediction and may thus make contribution to developing novel prognostic indexes of ccRCC. Furthermore, we analyzed the expression profiles of the model genes in the pathological grade and stage of RCC. The model-associated immune genes strongly showed an association with immuno-infiltrating cells, which may be used for targeting clinical immunotherapy.

However, our research still has limitations. First, our study only included the expression profiles of a part of the Western population. Extensive sample sequencing data from other countries and races are needed to enhance our conclusions. Second, our results were based on the RNA sequencing results of entire tumor tissue, and the diversity of different cell compositions in the TME was not considered. Third, we only focused on transcriptional expression profile data. Gene methylation level, mutation level, and other equally essential data in tumor progression were not considered. These data are pivotal to exploring tumor progression.

## Conclusion

The research provides a basis for the application of immune genes in the prognosis of ccRCC. It is credible for the immune gene risk score model to forecast the prognosis of ccRCC, which serves as an independent prognostic element for ccRCC patients. Our results may be helpful to in personalized treatment for patients with ccRCC and in exploring novel biomarkers for the targeted therapy of ccRCC.

## Supplementary Information

**Additional file 1: Figure S1.** PPI network constructed for the differences in expressing immune genes.

**Additional file 2: Figure S2.** Interrelation between 18 model immune genes and immune cell infiltration.

## Data Availability

The data is available in the ImmPort database (https://www.immport.org/) and The Cancer Genome Atlas (TCGA) database (https://cancergenome.nih.gov/).

## Abbreviations

RCC: Renal cell carcinoma
ccRCC: Clear cell renal cell carcinoma
TME: Tumor microenvironment
IRGs: Interferon-Responding Genes
TCGA: The Cancer Genome Atlas
KEGG: Kyoto Encyclopedia of Genes and Genomes
GO: Gene ontology
DAVID: Database for Annotation, Visualization, and Integrated Discovery
KIRC: Kidney renal clear cell carcinoma
GSEA: Gene enrichment analysis
FDR: False Discovery Rate
ROC: Receiver Operating Characteristic
LASSO: Least absolute shrinkage and selection operator
DEIGs: Differentially expressed immune genes
PPI: Protein-Protein interaction
AUC: Area Under Curve
OS: Overall Survival
K-M: Kaplan-Meier
PFS: Progression-free survival
