# Supplementary figures and images for: Identification of an independent immune-genes prognostic index for renal cell carcinoma
Source: BMC Cancer. 2021 Jun 29;21:746. doi: 10.1186/s12885-021-08367-6 (PMC8240194; doi:10.1186/s12885-021-08367-6)

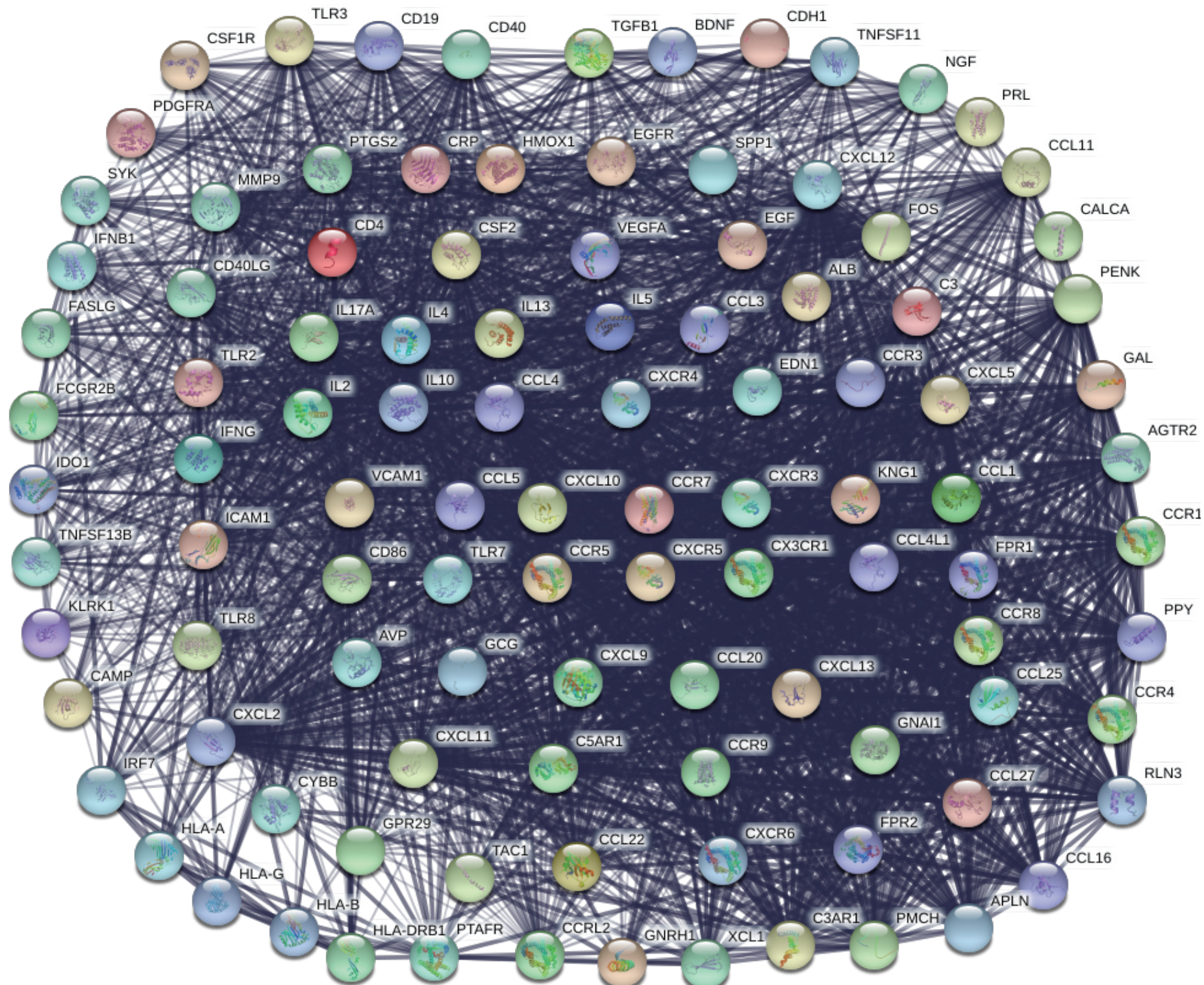

Supplement: Supplementary file 1 — Additional file 1: Figure S1. PPI network constructed for the differences in expressing immune genes. [file 12885_2021_8367_MOESM1_ESM.pdf]

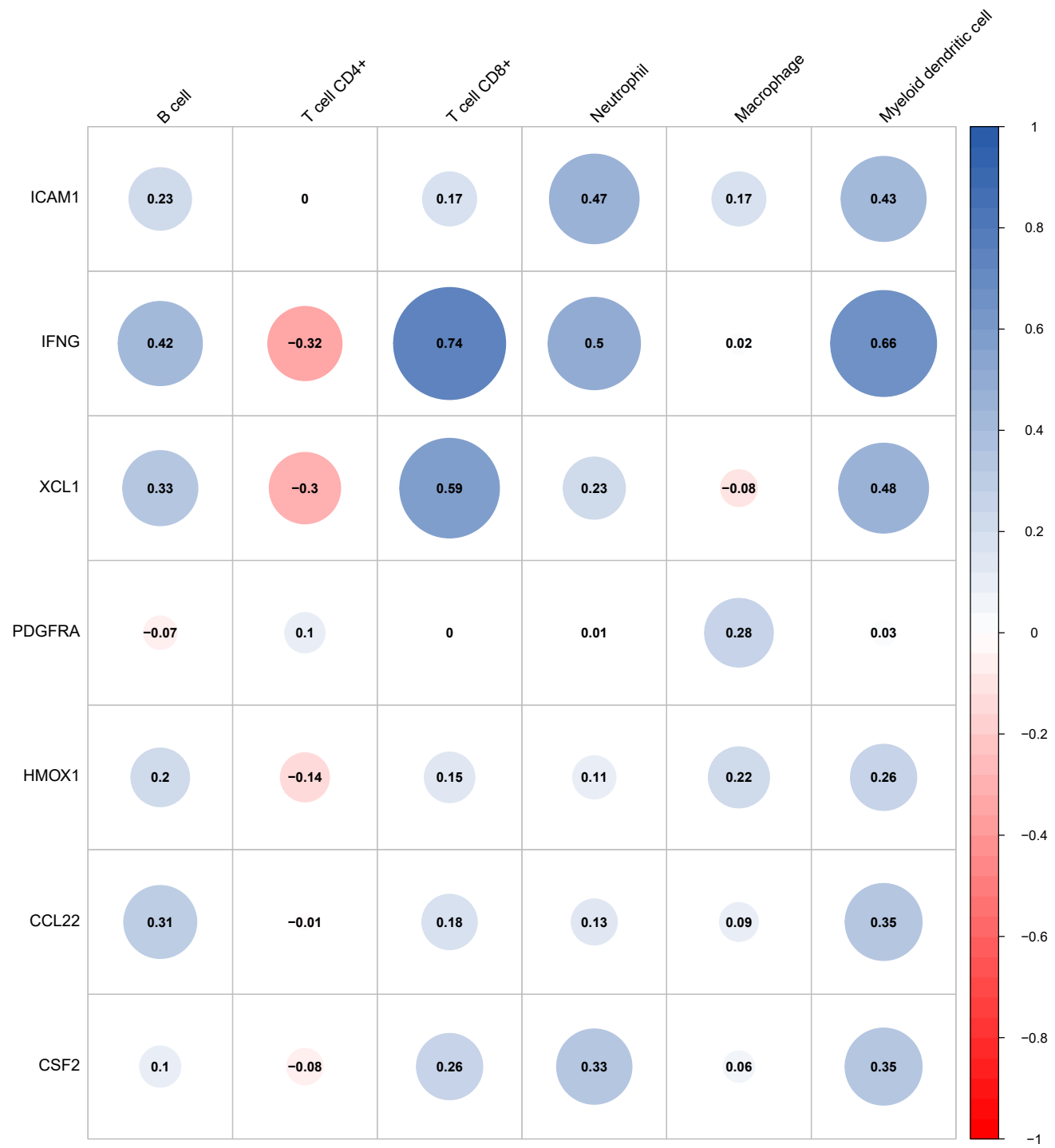

Supplement: Supplementary file 2 — Additional file 2: Figure S2. Interrelation between 18 model immune genes and immune cell infiltration. [file 12885_2021_8367_MOESM2_ESM.pdf]
